# Supplementary material for: Rethinking Measurement of Movement-Evoked Pain with Digital Technology
Source: medRxiv. 2025 Nov 26:2025.09.14.25335734. Originally published 2025 Sep 15. Preprint. [Version 2] doi: 10.1101/2025.09.14.25335734 (PMC12458538; doi:10.1101/2025.09.14.25335734)
Supplement: Supplement 1 [file media-1.pdf]

Rethinking Measurement of Movement-Evoked Pain with Digital Technology – Supplementary Materials

Madelyn R. Frumkin[1,2], Jingwen Zhang[3], Ziqi Xu[3], Salim Yakdan[4], Braeden Benedict[4], Saad Javeed[4], Justin Zhang[4,5], Kathleen Botterbush[4], Burel R. Goodin[6], Chenyang Lu[3], Wilson Z. Ray[4], & Jacob K. Greenberg[4]

[1]Center for Technology and Behavioral Health, Dartmouth College, Lebanon, NH, USA
[2]Department of Biomedical Data Science, Dartmouth College, Lebanon, NH, USA
[3]Department of Computer Science and Engineering, Washington University, St. Louis, MO, USA
[4]Department of Neurological Surgery, Washington University, St. Louis, MO, USA
[5]Department of Neurological Surgery, University of Utah, Salt Lake City, USA
[6]Department of Anesthesiology, Washington University, St. Louis, MO, USA

Fig S1. Impact of 6-minute step count thresholds on heart rate verification and participants included

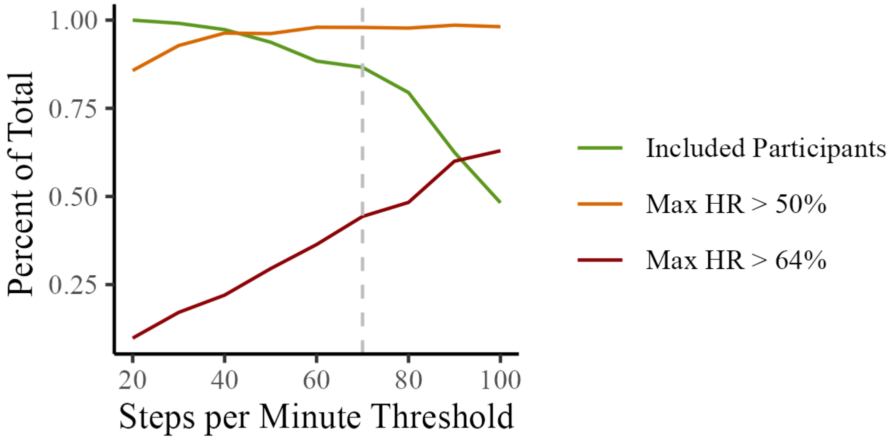

Note. A threshold of 70 steps per minute (grey dotted line) was retained as this threshold minimized participant exclusion (green line) due to a lack of observations, while maximizing heart rate (HR) verification of exercise intensity at 50% of max HR (orange line) and 64% of max HR (red line) thresholds.

Table S1. Reliability of MEP across step count thresholds

Steps per Minute Threshold	Absolute Post-Activity Pain		MEP Change Scores	
	M (SD) of Within-Person Variability	ICC	M (SD) of Within-Person Variability	ICC
50	12.23 (8.62)	0.72	13.64 (11.38)	0.04
60	11.6 (9.33)	0.74	13.35 (10.67)	0.05
70	10.14 (6.64)	0.76	11.58 (7.78)	0.08
80	10.76 (6.84)	0.76	12.49 (8.16)	0.06
90	10.66 (7.41)	0.74	11.58 (8.35)	0.07
100	12.5 (8.49)	0.68	13.23 (9.15)	0.11

Note. M = mean; SD = standard deviation; ICC = intraclass correlation coefficient

Table S2. Univariate predictors of post-activity pain ratings across step count thresholds

Steps per Minute Threshold	Average Pain	Lag-1 Pain	Survey	$Survey^2$	Amount of PA	Prior Activity	Time Lag
50	22.07 (0.48)*	3.58 (0.49)*	0.93 (0.33)*	-0.91 (0.28)*	0.14 (0.42)	0.30 (0.43)	-0.14 (0.52)
60	22.53 (0.55)*	2.89 (0.56)*	0.40 (0.38)	-0.87 (0.31)*	0.21 (0.47)	0.52 (0.48)	-0.61 (0.57)
70	22.46 (0.72)*	3.00 (0.62)*	0.43 (0.42)	-0.64 (0.35)	-0.04 (0.51)	0.45 (0.53)	-0.08 (0.62)
80	22.70 (0.79)*	2.47 (0.73)*	0.33 (0.50)	-0.90 (0.41)*	-0.23 (0.60)	0.58 (0.61)	-0.87 (0.75)
90	22.95 (0.88)*	1.96 (0.90)*	0.47 (0.60)	-1.00 (0.49)*	-0.33 (0.74)	0.37 (0.76)	1.23 (0.95)
100	22.93 (1.28)*	2.66 (1.37)	0.48 (0.81)	-0.80 (0.70)	0.41 (1.05)	1.36 (1.09)	0.92 (1.42)

Note. Model results are presented as coefficient (standard error). * = $p < .05$; PA = physical activity

Table S3. Multivariate predictors of post-activity pain ratings across step count thresholds

Steps per Minute Threshold	Average Pain	Lag-1 Pain	$Survey^2$	Amount of PA	Prior Activity	Time Lag
50	21.87 (0.50)*	3.99 (0.51)*	-1.07 (0.26)*	-0.30 (0.46)	0.54 (0.47)	-0.61 (0.52)
60	22.41 (0.59)*	3.27 (0.58)*	-1.00 (0.30)*	-0.28 (0.51)	0.86 (0.52)	-0.99 (0.58)
70	22.31 (0.76)*	3.31 (0.64)*	-0.63 (0.33)	-0.56 (0.57)	1.12 (0.58)	-0.42 (0.62)
80	22.43 (0.88)*	3.02 (0.76)*	-0.87 (0.39)*	-0.73 (0.67)	1.29 (0.70)	-1.00 (0.76)
90	22.73 (0.97)*	2.24 (0.93)*	-0.76 (0.46)	-0.64 (0.80)	0.91 (0.80)	0.64 (0.96)
100	22.65 (1.41)*	3.05 (1.30)*	-0.80 (0.62)	-0.63 (1.15)	1.71 (1.17)	0.50 (1.43)

Note. Model results are presented as coefficient (posterior standard deviation). * = 95% credible interval does not contain zero; PA = physical activity

Table S4. Univariate predictors of MEP change scores across step count thresholds

Steps per Minute Threshold	Average Pain	Lag-1 Pain	Survey	$Survey^2$	Amount of PA	Prior Activity	Time Lag
50	-0.20 (0.72)	-7.78 (0.53)*	-1.03 (0.71)	-0.80 (0.60)	0.59 (0.61)	1.25 (0.62)*	-2.44 (0.70)*
60	0.09 (0.88)	-8.64 (0.61)*	-1.79 (0.84)*	-0.55 (0.70)	0.34 (0.71)	2.13 (0.72)*	-2.39 (0.82)*
70	-0.53 (0.99)	-8.17 (0.66)*	-1.82 (0.92)*	0.23 (0.75)	-0.31 (0.77)	2.05 (0.78)*	-1.08 (0.86)
80	-0.07 (1.09)	-8.43 (0.78)*	-1.54 (1.11)	-0.49 (0.89)	-0.32 (0.92)	1.77 (0.92)	-1.74 (1.03)
90	1.34 (1.27)	-8.79 (0.94)*	-0.57 (1.38)	-0.28 (1.04)	-2.16 (1.13)	0.84 (1.12)	-0.38 (1.24)
100	2.25 (1.99)	-9.90 (1.43)*	1.16 (1.97)	-1.59 (1.51)	-3.36 (1.71)	0.10 (1.70)	-0.71 (1.80)

Note. Model results are presented as coefficient (standard error). * = $p < .05$; PA = physical activity

Table S5. Multivariate predictors of MEP change scores across step count thresholds

Steps per Minute Threshold	Average Pain	Lag-1 Pain	Survey	Amount of PA	Prior Activity	Time Lag
50	-0.19 (0.82)	-7.55 (0.53)*	-0.46 (0.53)	-0.19 (0.58)	0.91 (0.62)	-1.59 (0.67)*
60	0.13 (0.96)	-8.38 (0.62)*	-1.00 (0.60)	-0.18 (0.64)	1.43 (0.68)*	-1.00 (0.76)
70	-0.60 (1.09)	-7.96 (0.67)*	-0.88 (0.65)	-0.41 (0.69)	1.26 (0.74)	0.07 (0.79)
80	-0.17 (1.19)	-8.08 (0.79)*	-1.18 (0.76)	-0.72 (0.83)	1.11 (0.88)	-0.78 (0.98)
90	1.29 (1.41)	-8.51 (0.95)*	-0.30 (0.94)	-1.31 (0.95)	0.96 (1.03)	1.32 (1.23)
100	2.11 (2.26)	-9.39 (1.46)*	0.99 (1.39)	-1.46 (1.49)	0.43 (1.66)	-0.12 (1.81)

Note. Model results are presented as coefficient (posterior standard deviation). * = 95% credible interval does not contain zero; PA = physical activity

Table S6. Descriptives of resting pain ratings by pre-EMA activity levels.

Steps per Minute Threshold	Number of Observations	Percent of Total	Median (SD) Pain Rating	% Severe Pain
<1 spm	1667	31%	56.0 (27.7)	20.8%
1-5 spm	1732	32%	59.5 (27.0)	19.4%
5-10 spm	1030	19%	60.0 (25.9)	21.5%

Note. SD = standard deviation; severe pain cut-off = 80/100
